# The Best Splints for Bow Legs

**Published:** 1909-07-10

**Authors:** 


### THE BEST SPLINTS FOR BOW LEGS.

Dr. Arthur Todd-White has made public, in the form of a letter to the *Guy's Hospital Gazette*, some very pertinent remarks upon the question of bow-leg splints. He complains that the way of treating bow-legs still in vogue is antiquated, and he thinks it time that it gave place to a more rational method.

Dr. Todd White's own experience began with a boy, a relative, who had bow-legs, and whom he therefore took to an eminent "specialist," in addition to making personal inquiries at various hospitals as to the latest treatment. He found everywhere the same tale: "Put on long splints, and keep him off his feet."

This treatment, he contends, is entirely wrong in principle and does more harm than good. The disadvantages are many, the most important being that the beneficial effects of muscular action are done away with, the circulation in the limbs is interfered with, the splints are easily displaced, and have to be put on afresh very often, which takes time, the child is utterly miserable, the want of exercise is deleterious to its health, and the bandages get dirty.

The proper splints for bow-legs are made of poroplastic felt, are placed on the inside of the leg, extending from a little above the knee to the ground. They are fastened with three straps, one immediately below the knees and one round the ankle, over boots if the latter are worn. These two straps are not drawn very tight, and the third one, which goes round the calf and has a square leather pad on its outer side, is tightened every day until the limb is straight.

There is no need whatever, Dr. Todd-White states, to have a splint for the thigh, the femur being rarely affected, and the powerful muscles *with exercise* curing any deformity by themselves.

The advantages claimed for these splints are as follows: First, they absolutely cure the bow-legs; Dr. Todd-White after years of experience can guarantee this. Secondly, they permit of the child's running about and developing its muscles, the latter in their turn tending to draw the bones into a normal shape. Thirdly, they are easier to put on than a pair of boots, and therefore can be removed when the child lies down, or when massage is needed, or for sea-bathing. They can be kept clean; covered with brown leather they look like leggings; the children like them, the older ones putting them on themselves.

These splints are not new to the profession, but they have been overlooked. They cost about 15s., and they can be made by any instrument maker. Two pairs will last long enough to effect a cure—namely, about three years.

